# Confidence Intervals for fMRI Activation Maps

**DOI:** 10.1371/journal.pone.0082419

**Published:** 2013-12-02

**Authors:** Stephen A. Engel, Philip C. Burton

**Affiliations:** 1 Department of Psychology, University of Minnesota, Twin Cities, Minneapolis, Minnesota, United States of America; 2 Office of the Associate Dean for Research, University of Minnesota, Twin Cities, Minneapolis, Minnesota, United States of America; Cuban Neuroscience Center, Cuba

## Abstract

Neuroimaging activation maps typically color voxels to indicate whether the blood oxygen level-dependent (BOLD) signals measured among two or more experimental conditions differ significantly at that location. This data presentation, however, omits information critical for interpretation of experimental results. First, no information is represented about trends at voxels that do not pass the statistical test. Second, no information is given about the range of probable effect sizes at voxels that do pass the statistical test. This leads to a fundamental error in interpreting activation maps by naïve viewers, where it is assumed that colored, “active” voxels are reliably different from uncolored “inactive” voxels. In other domains, confidence intervals have been added to data graphics to reduce such errors. Here, we first document the prevalence of the fundamental error of interpretation, and then present a method for solving it by depicting confidence intervals in fMRI activation maps. Presenting images where the bounds of confidence intervals at each voxel are coded as color allows readers to visually test for differences between “active” and “inactive” voxels, and permits for more proper interpretation of neuroimaging data. Our specific graphical methods are intended as initial proposals to spur broader discussion of how to present confidence intervals for fMRI data.

## Introduction

Activation maps are the most popular method for displaying the results of an fMRI experiment. They typically show colored voxels on an image of the brain, with color indicating whether neural responses at that location differ between experimental conditions. To create such maps, software packages conduct statistical tests at each voxel, and color significantly active voxels to indicate either *p* values, effect sizes, or a statistic such as *F* or *t*. The colored voxels are then overlaid onto a gray-scale anatomical brain image. 

The spatial nature of activation maps seems to invite comparison between regions; when different voxels have different colors, it is natural to think about relationships between them. Valid comparisons between voxels cannot be made using typical activation maps, however, since the statistical test at each voxel (or cluster of voxels) is done independently, and comparisons require statistical tests that span multiple voxels. Activation maps are neither designed nor intended to support comparisons between voxels. Nevertheless, we believe that naïve map-readers make them.

**Figure 1:**
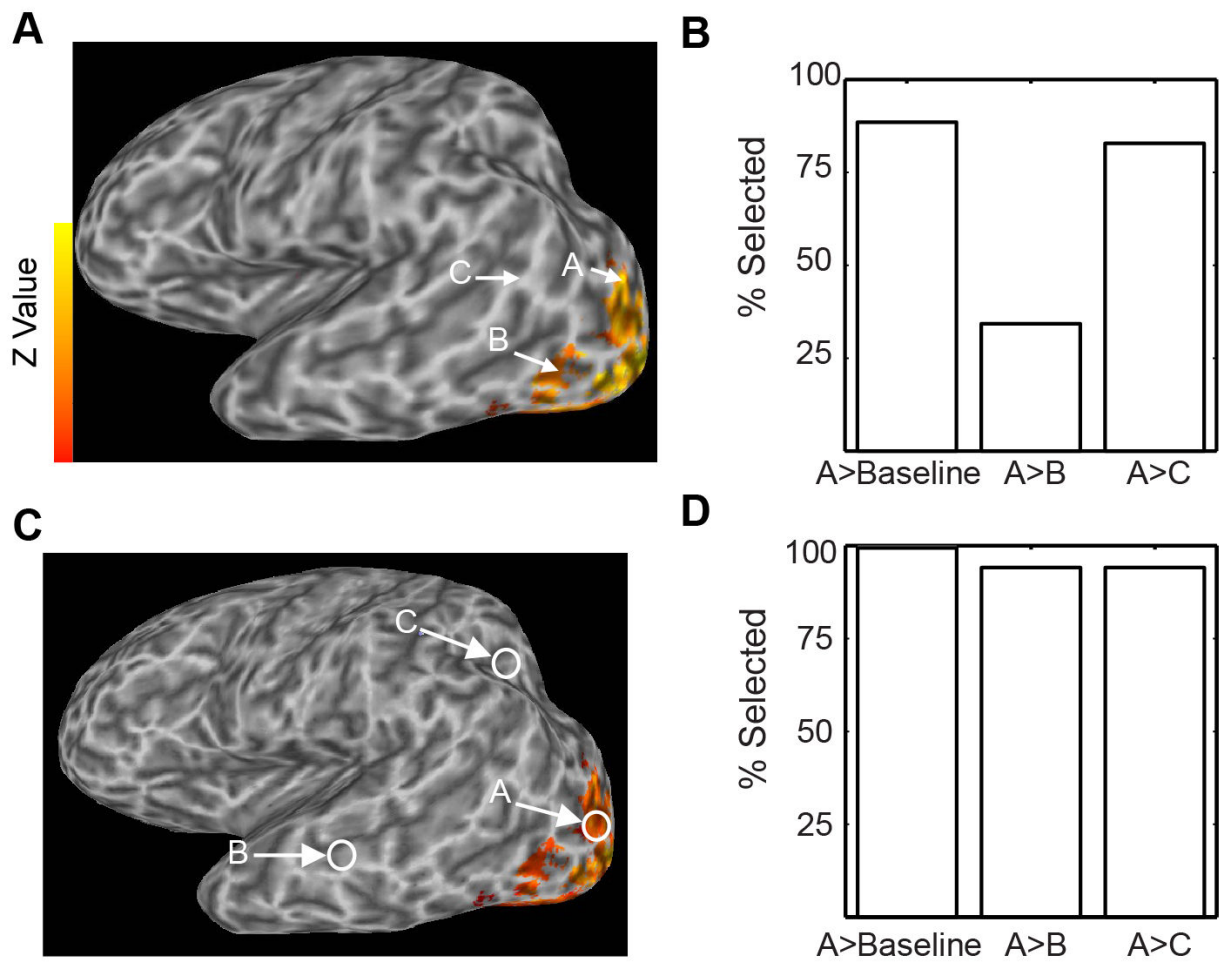
The fundamental error of interpreting brain maps. A. Brain activation map presented to Survey 1 respondents who were asked to compare points labeled “A,” “B,” and “C.” B. 83% of respondents believed the colored location “A” to be reliably more active than the uncolored location. C. Activity map presented to Survey 2 respondents. D. Over 90% of respondents believed the colored location to be reliably more active than the uncolored ones.

In particular, readers of activation maps often assume that significant, colored voxels differ reliably from nonsignificant, uncolored voxels. To illustrate this problem, consider Figure 1A, which shows activation in an experimental condition relative to a baseline condition. The map may make it appear that mean “activity” (the difference in signal between the condition of interest and baseline) at location B is significantly lower than activity at location A, since strong differences in color intuitively map to large differences in activation. This conclusion is not warranted, of course, since the means and variances of activity at voxels that are not reliably greater than zero, such as voxel B, are not displayed. It could well be that the mean activity at B is equal to or even greater than that at A, but that high variability prevents location B from showing a significant difference from zero. 

We believe that this fundamental error in interpreting activation maps is widespread within the nonexpert audience of neuroimaging studies. Furthermore, even experts often fail to report the statistical testing that could correct it [1,2]. The joint goals of this paper are to document the error and present possible solutions. 

Confidence intervals have proved to be an efficient method for correcting similar problems in other types of data (e.g., 3). Because they allow both comparisons between conditions (e.g. voxel activation) and baseline and between the conditions themselves, confidence intervals should be useful for helping readers avoid making the fundamental error in activation map interpretation. Here, we present three methods for depicting confidence intervals in fMRI activation maps. While none is perfect, they should serve as the basis for future development of methods for depicting confidence intervals in neuroimaging data. Our second and third methods were specifically designed to be effective for a naïve audience, and so we tested them on samples of undergraduates. We found that they reliably improved the ability of subjects to interpret activation maps.

## Results

### Survey 1 and 2

To test whether the fundamental error of interpretation was widespread in nonexpert populations, in Survey 1 we showed 35 undergraduates the image in Figure 1A and asking if they believed location A was significantly more active than location C. Eighty-three percent responded that they indeed believed this was the case (Figure 1B). While such a conclusion may in fact be true, it is not warranted given the information available in the map alone. To make sure that subjects understood uncertainty regarding differences was possible, we repeated the survey with more probabilistic language (as well as better indications of the voxels in question) using the map in Figure 1C. Language for both surveys is reproduced in Materials and Methods, below. Even when given the option of “Can’t tell with confidence”, 94% of the 17 subjects tested rated the response that location A was more active than location C as “Very likely (Greater than 95% chance it is true)”. In analyses of Surveys 2, 3, and 4, only this response was classified as an error.

Based upon informal discussion with colleagues in the field, we believe that this error is likely prevalent in other populations, including the popular press, and perhaps even graduate students and PIs with limited backgrounds in statistics. The fundamental error is a version of the “erroneous interactions in neuroscience” that have recently been well documented in the literature: A random sample of Neuroscience articles found many cases of “comparing several brain areas and claiming that a particular effect (property) is specific for one of these brain areas” without explicit tests for statistical interactions between areas [2]. In the case examined here, the effect is “activity” , and the unsupported claim of specificity is that the uncolored voxels are less active than colored ones. Two other papers have also noted that unsupported claims of interactions across brain areas are commonly made by investigators in the field [1,6]. However, no previous study to date has documented the error in naïve readers of brain maps, and the frequency at which it is made here greatly surpasses rates reported for investigators.

### Depiction of confidence intervals in activation maps: Method 1

To reduce the prevalence of the fundamental interpretation error, we developed a way of showing confidence intervals in fMRI images. We first illustrate this idea using data from a simple visual experiment; the study compared activity measured when subjects viewed dynamic white noise to a baseline condition of activity measured when subjects viewed a uniform gray screen. 

**Figure 2:**
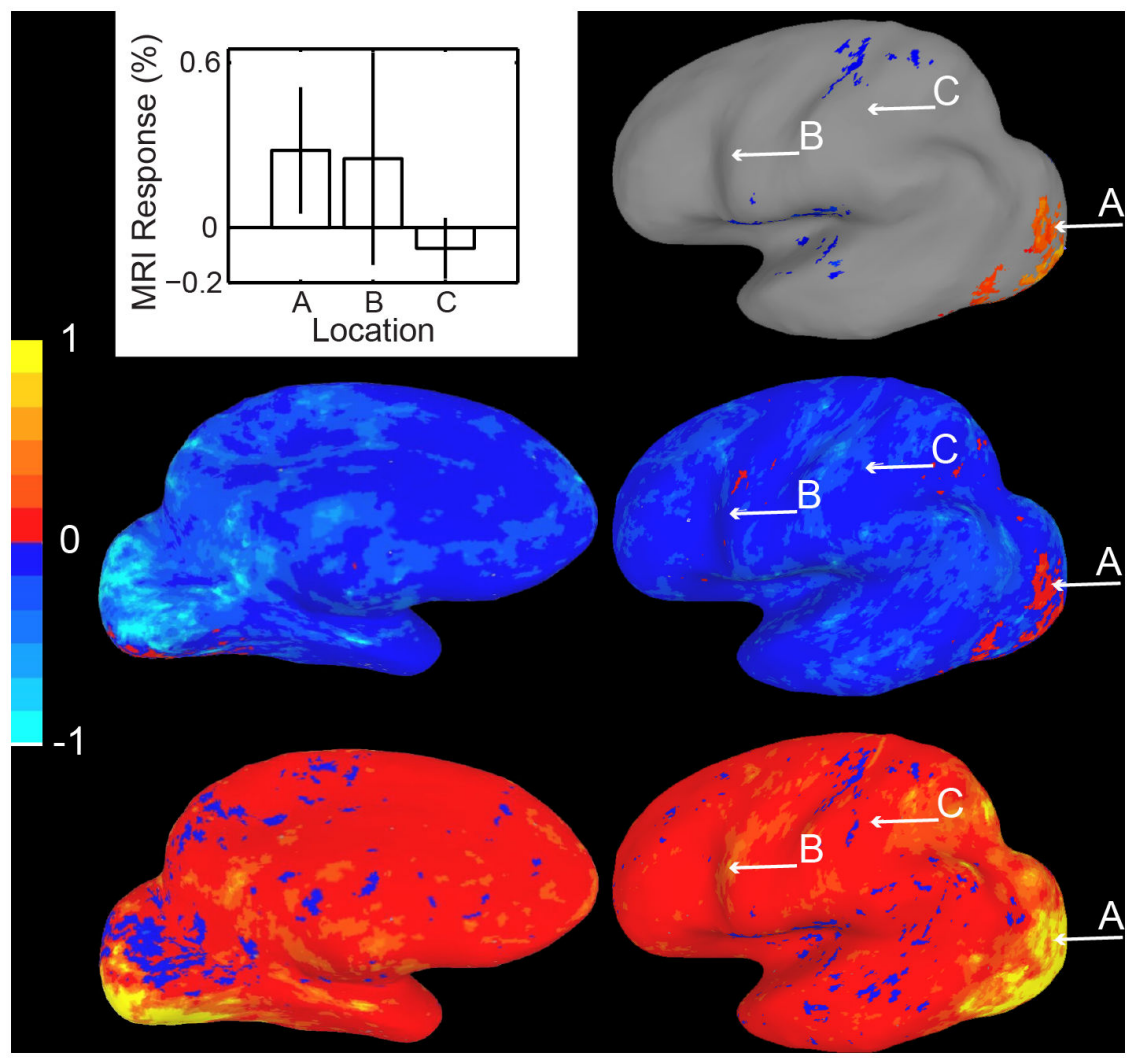
Confidence interval map Method 1, Data Set 1. Top, right. Conventional activation map for Data Set 1, visual stimulation vs. baseline. Top, left. Activation and confidence intervals for the three labeled locations at right are plotted. Middle row. Map of the lower bound of the 99% confidence interval for activation of each voxel. Bottom row.Map of the upper bound of the 99% confidence interval for each voxel. That locations A and B do not reliably differ can be seen by noting that the upper bound of point B (yellowish in lower panel) is higher than the lower bound of point A (reddish in middle panel).

Figure 2 shows the results of our method for constructing confidence intervals on activation maps. The upper right panel displays the group activation map on an inflated brain in Talairach space. Voxels where response to visual noise was significantly greater than baseline are colored, with the color reflecting activation in percent signal change. Activity at uncolored voxels is of course unspecified in these images, and the bar plot at the upper left shows that while one uncolored voxel (labeled C) is indeed reliably less active than a colored voxel (labeled A), another is not (labeled B). The middle and lower panels show the upper and lower boundaries, respectively, of 99% confidence intervals computed for the activation of each voxel. Color again reflects percent signal change, and positive values are plotted in the red-yellow range and negative values in the blue-cyan range. The size of the interval corresponds to the same alpha (p < 0.01) used to threshold the original activation map, which in both cases helps reduce Type 1 errors associated with multiple comparisons. Depending on the purposes of the confidence intervals, other choices of alpha would be possible. 

The image clearly shows that the active area is not likely to be significantly more active than several other regions scattered throughout the brain. These are regions whose upper confidence bound is greater than the lower confidence bound of the active region. Specifically, since the lower bound of the active region is reddish (as seen in the middle right panel), voxels whose upper bounds are yellowish do not significantly differ from the active region. For example, location B is yellowish in the lower panel, which displays the upper bound. As can be verified in the bar graph, its activity does not differ reliably from the “active” voxel A. Location C is an example voxel whose activity does reliably differ from location A’s.

**Figure 3:**
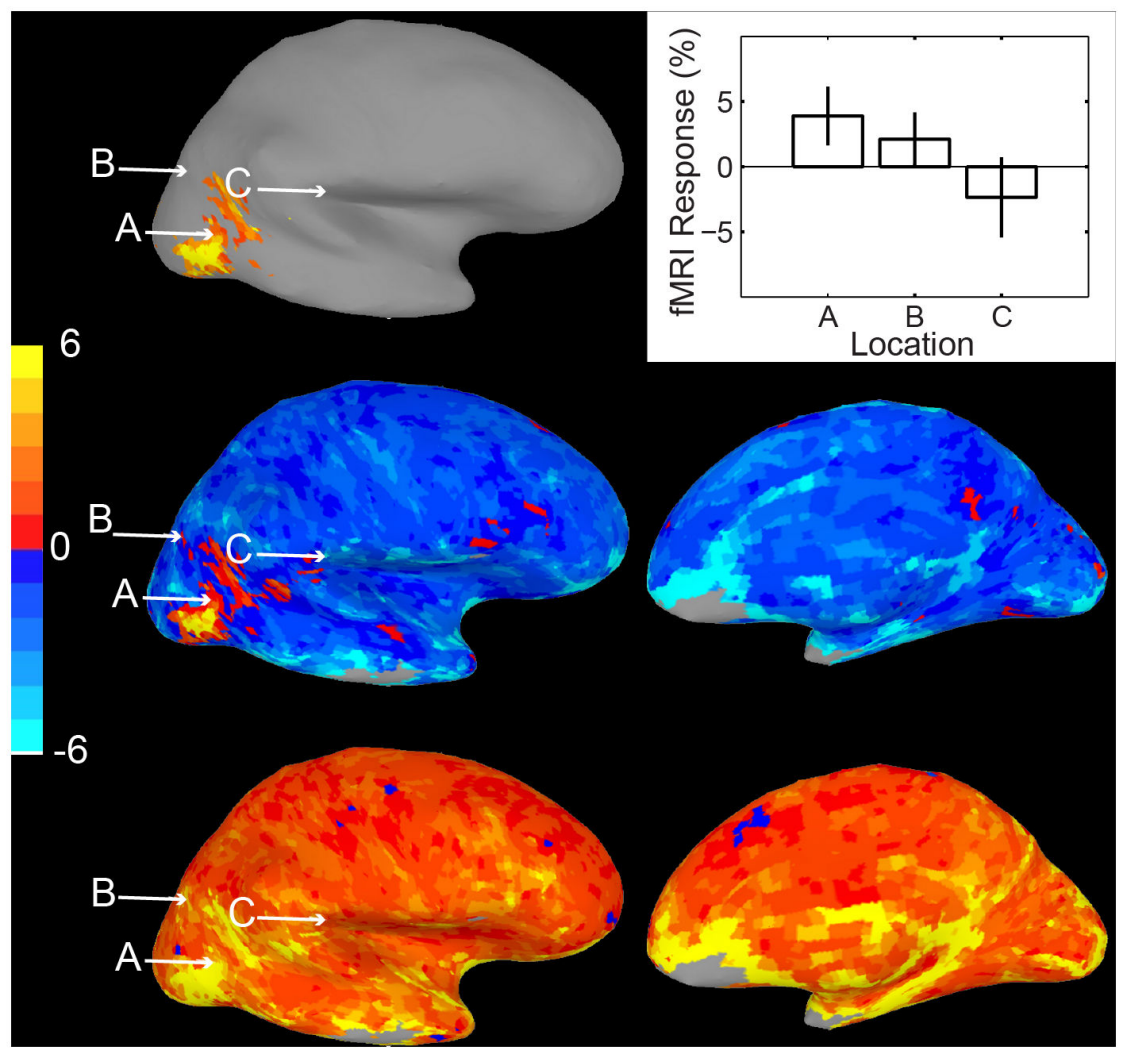
Confidence interval map Method 1, Data Set 2. Top, left. Conventional activation map for Dataset 2, human motion vs. nonhuman motion. Top, right. Activations and confidence intervals for the three labeled points. Middle row. Map of the lower bound of the 99.9% confidence interval for activation of each voxel. Bottom row. Map of the upper bound. Again, the lack of a difference between locations A and B can be seen by comparing the color of the upper bound of B to the lower bound of A.

Figure 3 shows a similar analysis for a second data set. In this case, the standard activation map (top) shows voxels where viewing human motion produced reliably greater activity than viewing motion of tools. Comparing the lower bound (middle panels) and upper bound (lower panels) images for a 99.9% confidence interval reveals that for several “inactive” cortical locations response does not reliably differ from the “active” cortical locations. For example activity at location A does not differ from activity at location C. For these voxels, as well as voxel B where activity does in fact differ from A, activation values and confidence intervals are again shown in a conventional bar plot in the upper panel.

The images of confidence interval bounds in Figures 2 and 3 map relatively closely onto the bounds presented with error bars in traditional univariate bar graphs. However, identifying significant differences among voxels is difficult because it involves scanning two separate images, and because non-overlapping upper and lower confidence intervals may fall within the same color category. For example it is difficult to tell whether activity in a red voxel in the middle panels of Figure 2 is slightly higher than in a red voxel in the lower panels. An additional problem for naïve viewers is that such comparisons require understanding the relatively technical concept of upper and lower bounds of confidence intervals, and keeping track of which is which while scanning the two images.

### Depiction of confidence intervals in activation maps: Methods 2 and 3

**Figure 4:**
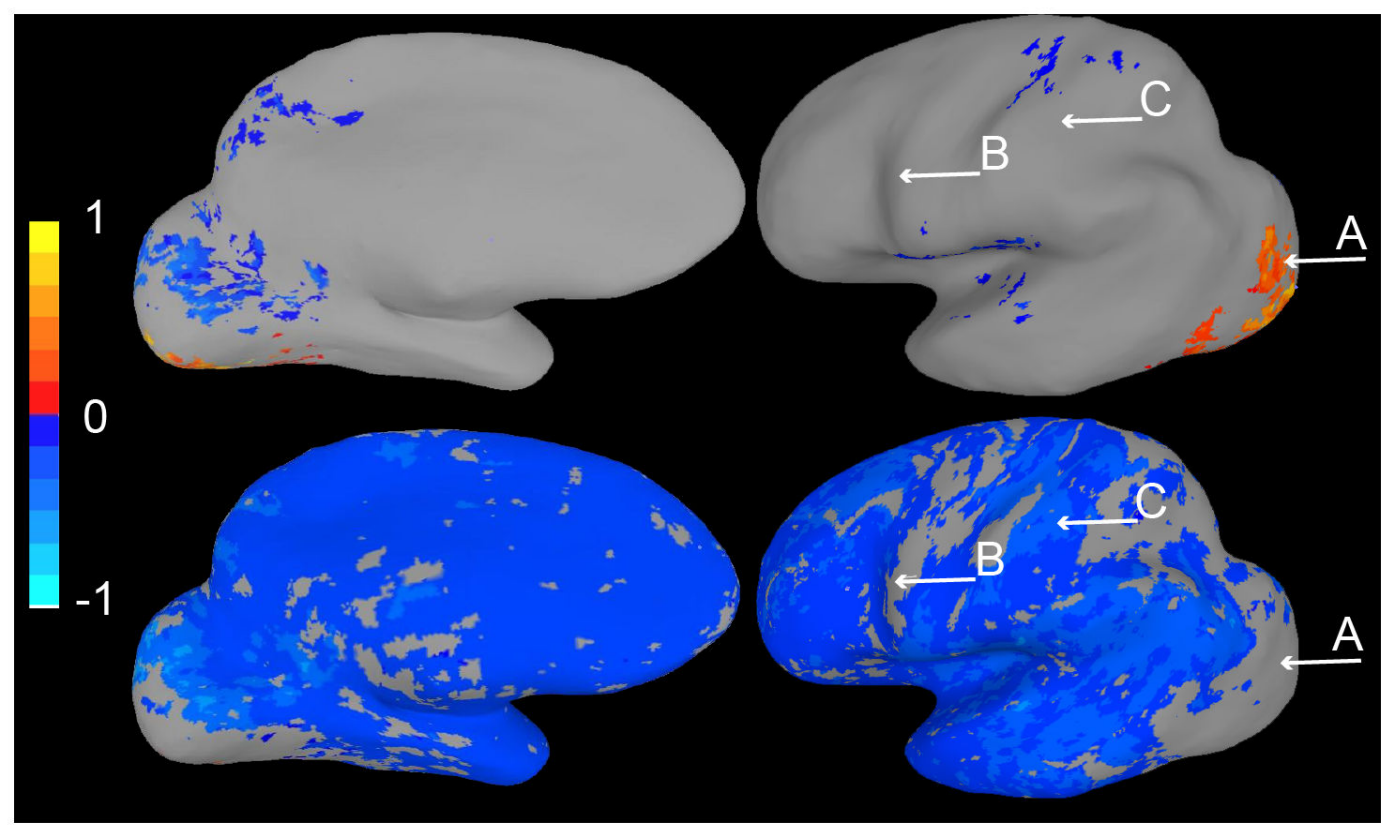
Confidence interval map Method 2, Data Set 1. Top. Conventional activation map, as in Figure 2. Bottom. Results of comparing activity within the active ROI to each voxel in cortex are shown. Only voxels that differ reliably from the ROI are colored. Voxels that do not differ reliably are uncolored.

**Figure 5:**
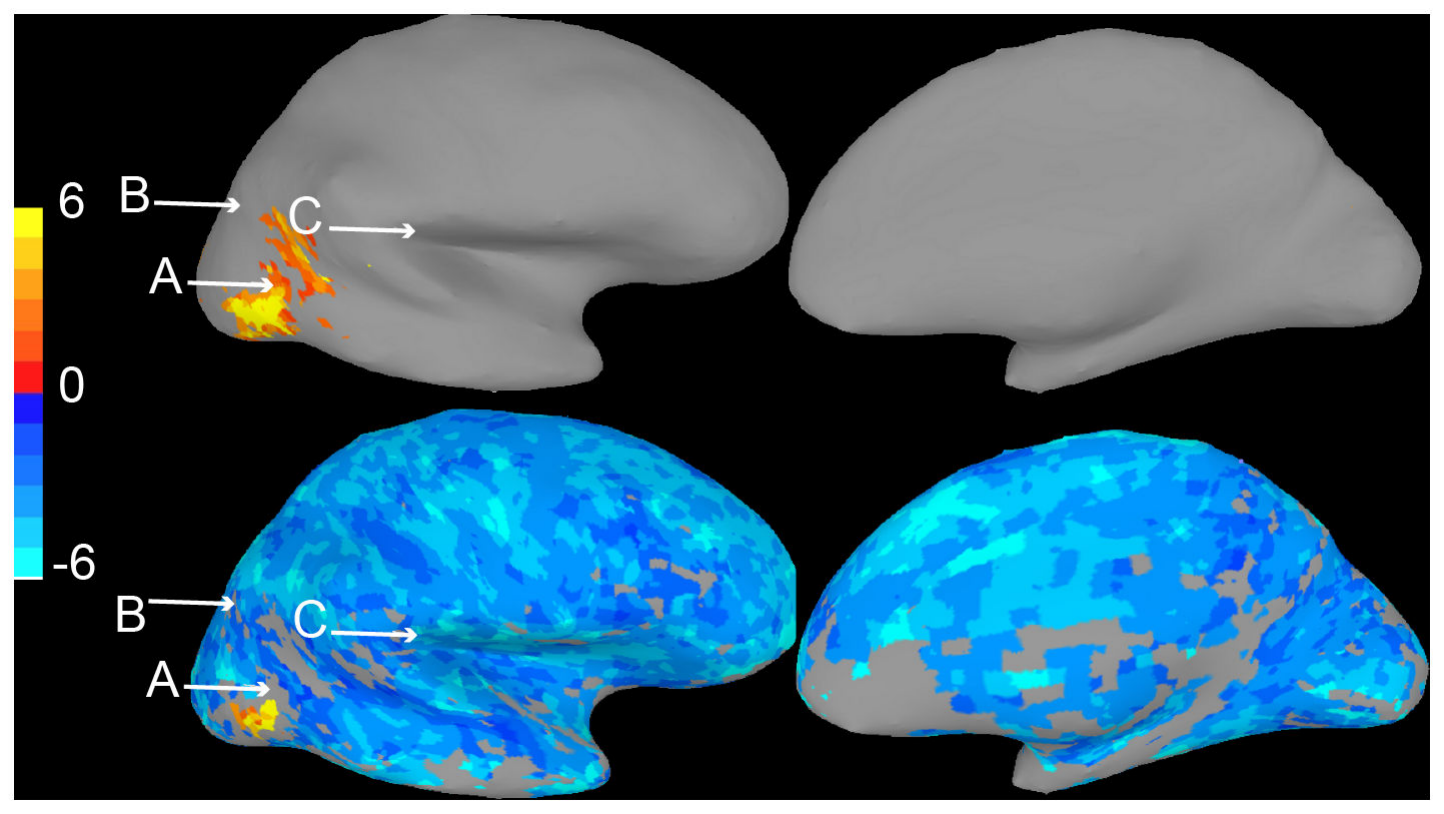
Confidence interval map Method 2, Data Set 2. Activation map (top) and results of comparison to ROI (bottom) for data set 2, with conventions as in Figure 4.

An alternative approach colors voxels according to whether they differ from a given region of interest. We chose as our region the active occipital voxels in Figure 4 (top), replotted from Figure 2. As a simple test of whether voxels outside the region differ from it, we chose to compare each voxel to a representative one from the ROI, in this case the voxel that was at the 75^th^ percentile of activity level within the region (which is close in value to the voxel labeled “A”). Each voxel in the brain was compared with the representative one using a standard t-test. Voxels that were significantly less activated than the ROI reference voxel were colored with the blue/cyan range of the color scale, while those that were significantly greater were colored in the red/yellow range. Figure 4 shows results of this analysis for Data Set 1 while Figure 5 shows the analysis for Data Set 2. Arrows depict the same sample voxels from previous figures that do and do not differ significantly from each other, with activity levels as shown in the bar graphs in Figures 2 and 3. 

The method clearly identifies voxels whose activity is reliably lower than the ROI’s; such voxels are colored blue. Voxels whose activity is not reliably lower are also easy to identify. Such voxels are uncolored or red.

**Figure 6:**
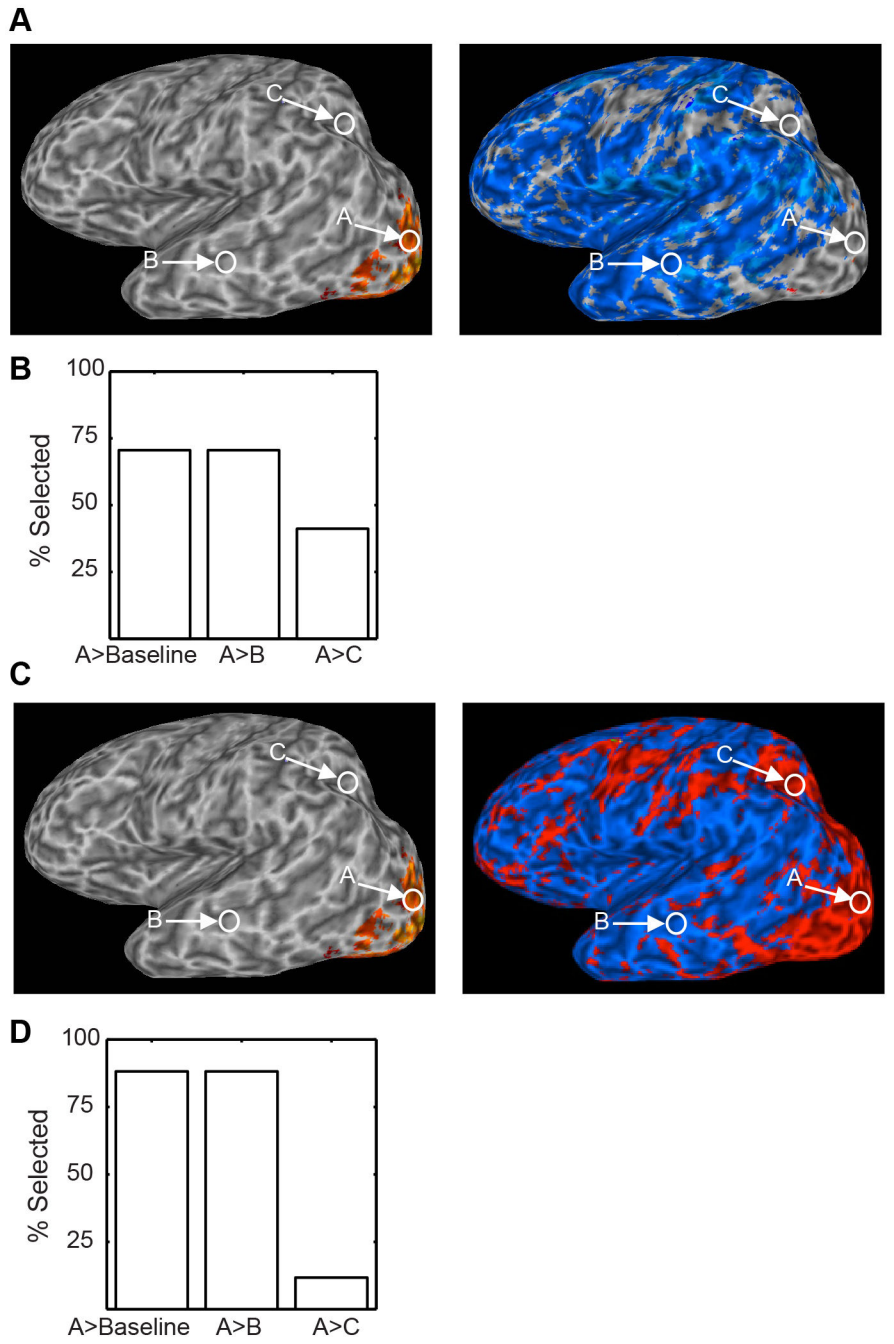
Survey images and results for confidence interval Methods 2 and 3. A. Brain activation maps presented in Survey 3. B. Fewer respondents believed the colored location (A) in the basic activation map was reliably more active than the uncolored one (C), when presented with a Method 2 display. However, a substantial number of respondents were not certain that the blue location (B) were reliably less active than the ROI. C. Brain activation maps presented in Survey 4. D. Very few respondents believed the colored location in the basic activation map (A) was reliably more active than the uncolored one (C), and most respondents identified the blue location (B) as reliably less active than the colored ones in the basic map (A).

Survey 3 demonstrated the effectiveness of this method (Figure 6A, B). The number of subjects making the fundamental error, tested by comparing activity at locations A and C, dropped significantly from 94% to 41% (Chi-square test, as are all that follow, *p < 0.01*). While an improvement, this error rate remains remarkably high, which shows how strongly subjects believe a lack of coloring corresponds to a lack of activity. In addition, some subjects may have interpreted the blue coloring as positive “activity” in and of itself, which may have reduced the number of correct responses for the question comparing activation in the ROI with a blue region (comparing activity at locations A and B).

**Figure 7:**
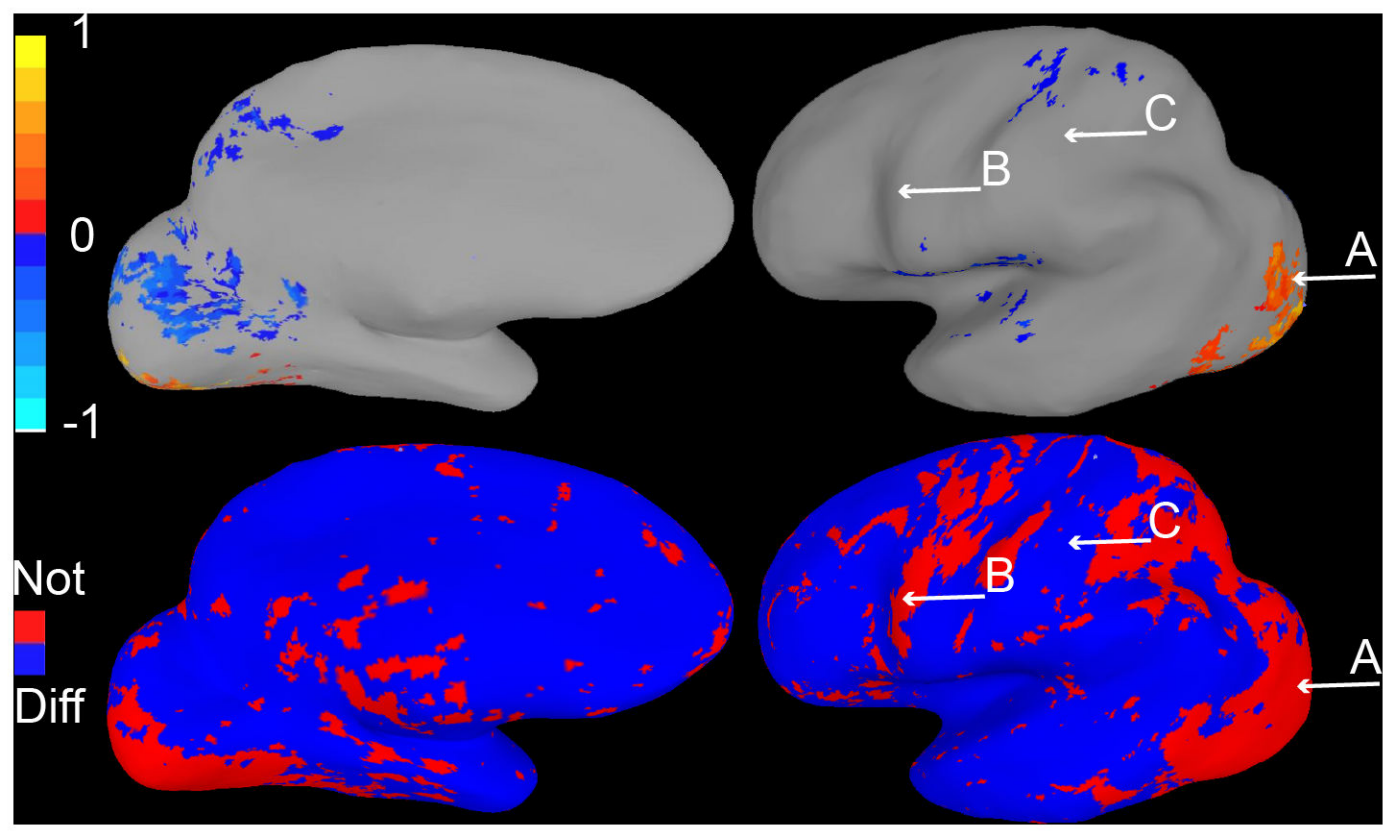
Confidence interval map Method 3, Data Set 1. Top. Conventional activation map, as in Figure 2. Bottom. Results of comparing activity within the active ROI to each voxel in cortex are shown. Voxels that differ reliably from the ROI are colored blue. Voxels that do not differ reliably are colored red.

**Figure 8:**
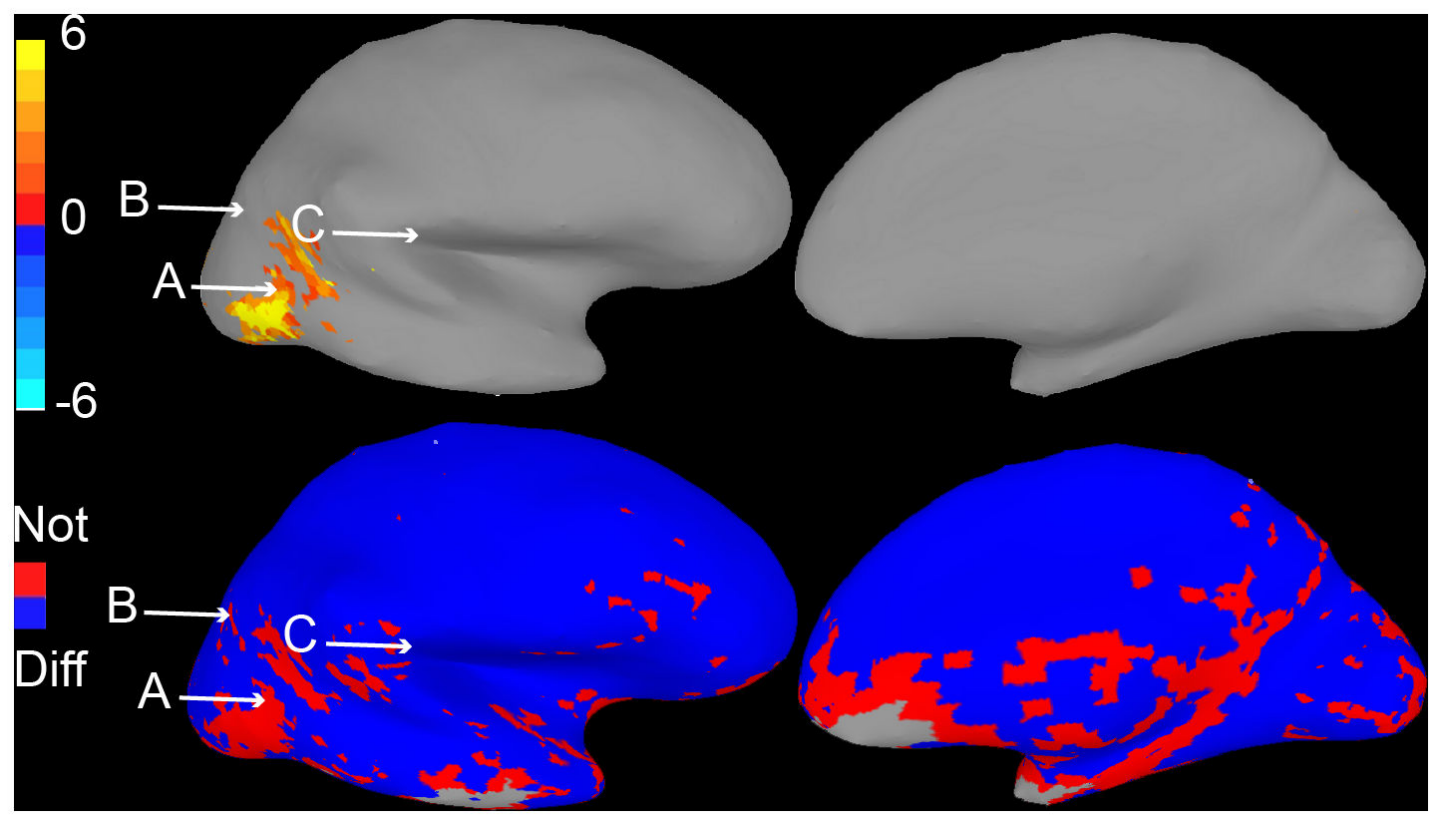
Confidence interval map Method 3, Data Set 2. Activation map (top) and results of comparison to ROI (bottom) for Data Set 2, with conventions as in Figure 7.

To better match intuitions mapping color to activity, and to simplify the display even further for a naïve audience, we developed a third type of display. In it, voxels that significantly differ from the representative ROI voxel simply colored blue, and all voxels that do not are simply colored red. Figures 7 and 8 illustrate this type of display for our two data sets.

This method proved to be very effective in correcting the fundamental interpretation error: it was tested in Survey 4, and only 12% of subjects made the error, a significant improvement over our initial surveys (*p < 0.001*; Figure 6C, D). Method 3 also helped subjects to determine which areas were in fact different from the ROI raising it to 88% (from 71% with Method 2). Overall, the number of errors made across all three questions was significantly lower in Survey 4 than Survey 3 (*p < 0.05*).

## Discussion

Naïve viewers almost universally misinterpret maps of brain activity, by assuming that colored regions are reliably more active than uncolored ones. This fundamental error is a specific form of one commonly made across neuroscience, inferring a statistical interaction in the absence of a test [1]. We presented three methods to encourage more accurate reading of brain maps, based upon displaying confidence intervals for neural activation. Our second and third methods were tested on naïve subjects, and greatly reduced the prevalence of interpretation errors.

The advantage of our Method 1 is the completeness with which it displays the confidence intervals. Its disadvantages are its conceptual complexity, and that comparing colors across cortical locations can be imprecise. For the studies with multiple subjects, the method also has another difficulty. When comparing two voxels in a standard GLM analysis, the relevant error term is the standard error of the difference between the voxels. Using this term effectively factors out between subjects variance in overall activity levels. However, our confidence intervals include such variance, and it is unclear how to remove it from brain maps. In behavioral experiments, between subjects variance can be removed from error bars either by normalizing each subject’s scores, by using the error term from a subject by condition analysis of variance [3], or by plotting error bars for pairwise differences between conditions (the last method is probably the best, see 7). None of these is likely to be effective for imaging data, however, because the conditions being compared are voxels, and the population of voxels is so large and diverse.

Methods 2 and 3 have the advantage that they make visually clear which regions in the brain do, and do not, differ from an ROI. Their disadvantage is that they can display results for only a single ROI at a time. One way to overcome this problem would be to show multiple maps, which might be appropriate for the many neuroimaging papers that focus their analysis on only one or two key ROIs. 

Method 3 may seem heavy-handed, since it explicitly informs readers which voxels are, and are not, reliably less active than an ROI. Such explicitness seems necessary, however, as method 2, which only colored voxels that were reliably less active than the ROI, produced less accurate interpretations in our survey. Because of its simplified color map, Method 3 may also be displayable in a relatively small format, perhaps as an inset of figures showing primary activation maps. However, the fact that Method 3 colors red some voxels that do not show activity in the primary maps may lead readers to faultily assume that they are in fact active.

In our opinion, the best approach would be to make use of a dynamic display. An interactive tool could allow a user to select a reference ROI off of a standard activation map. The tool could then automatically generate and display a confidence interval image using either Method 2 or 3. Such interactive displays could be an attractive part of standard analysis packages, and could be incorporated into electronic journals and as online supplemental material for paper publications. This approach would also overcome our somewhat arbitrary choice of activity of the voxel in the 75^th^ percentile of activation as representative of the ROI; other voxels could be selected, and a number of options for ROI comparison could be presented. 

The goal of this paper was to document the need for confidence intervals in neuroimaging research and to demonstrate some proof-of-concept solutions. Our specific methods for computing and displaying confidence intervals could be improved, however. Most importantly, computing the lower and upper bounds on a purely voxel by voxel basis ignores statistical information present in clusters of voxels. It should be possible to develop methods to take this information into account while computing the upper and lower bound images. It may also be possible to develop better graphical methods for allowing confidence intervals to be displayed across voxels. Placing error bars on data maps is a problem that extends far beyond fMRI, and any solution that is developed could have wide applicability.

Misinterpreting “inactive”, i.e. uncolored, parts of activation maps is likely to be highly prevalent in the general public, and so is a major problem for the field. While the precise format may be improved further, we urge the field to solve this problem by adopting some method of displaying confidence intervals, and look forward to their eventually becoming a standard part data presentation.

## Materials and Methods

### Surveys

In four survey experiments, we tested undergraduate students participating in the Psychology Department subject pool. The research protocol was approved by the Office for Protection of Research Subjects at the University of Minnesota, and we obtained written informed consent from all subjects.

In Survey 1, 35 subjects viewed the map shown in Figure 1A, and were given the following written instructions:

The image above shows the results of a study that measured brain activity using functional MRI. The colors on the image show areas that were more active when subjects looked at pictures of faces compared to when they looked at a blank screen. Which of the following is true? (Circle all that apply.)

1. Areas A and B were significantly more active in response to faces than to a blank screen.
2. Area A showed significantly greater activity in response to faces than area B.
3. Area A showed significantly greater activity in response to faces than area C.

In Survey 2, a group of 17 subjects was shown the map in Figure 1C. This survey had voxels indicated both with arrows and circles, and the added caption “Parts of the brain that are more active (p < 0.05) when people look at faces than a blank screen are colored.” Instructions were reworded as follows:

The image above shows the results of an experiment that measured brain activity while people were looking at faces. Please look over both it and its caption now.

Use the image to answer the following. Circle the best answer for each question**.**

Is Area A active when people look at faces?

1. Very likely (Greater than 95% chance it is true)
2. Very unlikely (Less than 5% chance)
3. Can’t tell with confidence (between 5% and 95% chance)

Is Area A more active than area B when people look at faces?

1. Very likely (Greater than 95% chance it is true)
2. Very unlikely (Less than 5% chance)
3. Can’t tell with confidence (between 5% and 95% chance)

Is Area A more active than area C when people look at faces?

1. Very likely (Greater than 95% chance it is true)
2. Very unlikely (Less than 5% chance)
3. Can’t tell with confidence (between 5% and 95% chance)

In Survey 3, 20 subjects were shown both the maps in Figure 6A. The left image was identical to that used in Survey 2 and had the identical caption. The right image had the caption: “Area A is more active (p < 0.05) than areas colored bluish when people look at faces”. Subjects were then given identical instructions and questions as were given to those who participated in Survey 2.

In Survey 4, 17 subjects viewed the two maps shown in Figure 6C. The left figure and caption were identical to those used in the previous two surveys. The right figure had the caption: “Area A is more active (p < 0.05) than areas colored bluish when people look at faces. Activity in reddish areas is not statistically different from A”. The rest of the Survey was identical to the corresponding part of Survey 3.

### fMRI data analysis

To develop examples of fMRI confidence intervals, portions of existing data from one published and one unpublished study were reanalyzed, using standard GLM analysis methods to compute voxel by voxel statistics. Upper and lower 99% confidence intervals (dataset 1) and 99.9% confidence intervals (dataset 2) were then computed at each voxel and color-coded such that differences among voxels could be compared. 

Confidence intervals were computed using the standard formula: *CI =* μ *± t*(*crit*)*σ/sqrt(*n-1*), where μ is the mean percent signal change, *t*(*crit*) is the critical t value for the confidence range and data degrees of freedom, and *σ/sqrt*(*n-1*) is the standard error of the mean voxel activity.

Dataset 1 included data from 10 subjects performing a visual cortex localizer task as part of a broader unpublished study of vision (Olman C., et al.). Subjects viewed dynamic white noise patterns in 20 sec blocks separated by 16 sec of fixation on a gray field. Data were acquired from a 3T Siemens Tim Trio Scanner. EPI data were acquired in two runs of 134 volumes each that lasted 4 minutes and 28 seconds (TR=2000 ms, TE=28 ms, flip angle =90). Each EPI volume consisted of 34 3 mm slices (in-plane resolution 3 x 3 mm). 

Dataset 2 consisted of one subject’s data from a paper published in [4] that are available as part of a tutorial dataset at http://afni.nimh.nih.gov (and fMRI Data Center Archive Ascension No. 2-2003-113QA). The study investigated brain regions responsive to human and nonhuman motion. Stimuli were movies of human motion and moving tools, and point light displays of human motion and moving tools, and were presented in an event-related fashion with a jittered intertrial interval (minimum 3 seconds). Data were acquired in 10 runs of 4:36. For additional details about MR data and acquisition parameters, see 4.

For both data sets, standard preprocessing steps including six-parameter motion correction, slice-time correction, and spatial smoothing (4mm FWHM Gaussian blur) were performed. The general linear model (GLM) was used to estimate beta weights and conduct associated significance tests for effects of interest. These operations were conducted in AFNI [5].

For Dataset 1, beta weights for the visual noise vs. baseline contrast were transformed to a standard anatomical space (MNI), and a one-sample across-subjects t-test was conducted on beta weights with subjects (n=10) as a random factor. Data were thresholded at *p < 0.01* and a minimum cluster size of 1,000 μL. For Dataset 2, beta weights and associated t-tests were computed for a fixed-effect (single subject) contrast between human motion and tool motion. Data were collapsed across the movies vs. points variable in order to focus on a single, robustly activated contrast, and were thresholded at *p < 0.001*, and a minimum cluster size of approximately 984 μL. Because Dataset 2 consisted of a single subject, its fixed effect analysis was less conservative than the random effect analysis conducted with Dataset 1. Accordingly, a more conservative threshold was used with Dataset 2 to make the analyses comparable.
